# Influence of Social Media Platforms on Public Health Protection Against the COVID-19 Pandemic via the Mediating Effects of Public Health Awareness and Behavioral Changes: Integrated Model

**DOI:** 10.2196/19996

**Published:** 2020-08-19

**Authors:** Hani Al-Dmour, Ra’ed Masa’deh, Amer Salman, Mohammad Abuhashesh, Rand Al-Dmour

**Affiliations:** 1 The University of Jordan Amman Jordan; 2 Princess Sumaya University for Technology Amman Jordan

**Keywords:** social media platforms, Interventions, public health, awareness, public health protection, coronavirus, COVID-19, pandemic, behavioral change, Jordan, behavior, social media

## Abstract

**Background:**

Despite the growing body of literature examining social media in health contexts, including public health communication, promotion, and surveillance, limited insight has been provided into how the utility of social media may vary depending on the particular public health objectives governing an intervention. For example, the extent to which social media platforms contribute to enhancing public health awareness and prevention during epidemic disease transmission is currently unknown. Doubtlessly, coronavirus disease (COVID-19) represents a great challenge at the global level, aggressively affecting large cities and public gatherings and thereby having substantial impacts on many health care systems worldwide as a result of its rapid spread. Each country has its capacity and reacts according to its perception of threat, economy, health care policy, and the health care system structure. Furthermore, we noted a lack of research focusing on the role of social media campaigns in public health awareness and public protection against the COVID-19 pandemic in Jordan as a developing country.

**Objective:**

The purpose of this study was to examine the influence of social media platforms on public health protection against the COVID-19 pandemic via public health awareness and public health behavioral changes as mediating factors in Jordan.

**Methods:**

A quantitative approach and several social media platforms were used to collect data via web questionnaires in Jordan, and a total of 2555 social media users were sampled. This study used structural equation modeling to analyze and verify the study variables.

**Results:**

The main findings revealed that the use of social media platforms had a significant positive influence on public health protection against COVID-19 as a pandemic. Public health awareness and public health behavioral changes significantly acted as partial mediators in this relationship. Therefore, a better understanding of the effects of the use of social media interventions on public health protection against COVID-19 while taking public health awareness and behavioral changes into account as mediators should be helpful when developing any health promotion strategy plan.

**Conclusions:**

Our findings suggest that the use of social media platforms can positively influence awareness of public health behavioral changes and public protection against COVID-19. Public health authorities may use social media platforms as an effective tool to increase public health awareness through dissemination of brief messages to targeted populations. However, more research is needed to validate how social media channels can be used to improve health knowledge and adoption of healthy behaviors in a cross-cultural context.

## Introduction

### Background

Infection with coronavirus disease (COVID-19) has become a severe public health issue worldwide. COVID-19 is caused by severe acute respiratory syndrome coronavirus 2 (SARS-CoV-2), a novel coronavirus that recently emerged from China. In March 2020, the World Health Organization (WHO) declared that COVID-19 can be characterized as a pandemic. Therefore, it is of utmost importance to prevent further spread of the pandemic in public and health care settings [1,2]. Scholars have reported that evidence of the impact of social media on health knowledge, behavior, and outcomes show that these tools can be effective in meeting individual and population health needs. Most research addresses specific interventions and approaches, which vary widely in focus, target population, theoretical foundations, mode of delivery, functionality, and usability. Due to this wide variation, it is difficult to discover what works and how, and efforts to compare approaches are complicated [3]. General strategies and guidelines include social distancing, testing every suspected case, staying home, avoiding social gatherings, treating patients, and contact tracing [4]. However, some countries are taking stricter measures to contain the pandemic, such as lockdowns and mass testing.

Jordan has been under nearly total lockdown since March 14, 2020. After only a few COVID-19 cases appeared around the country, the Jordanian government took extraordinary measures, including implementing strict emergency laws. Travel restrictions were imposed on passengers in Jordan; all unauthorized travel into and out of the country and between cities was halted. Nonpharmaceutical physical distancing interventions, such as extended university and school closures and workplace distancing, were introduced to reduce the impact of COVID-19. Public awareness and prevention of COVID-19 infection play important roles in disease control; a lack of reasonable knowledge of infectious diseases leads to low detection rates. Therefore, to stop the spread of COVID-19 infection in Jordan, the Jordanian Ministry of Health launched specific national disease control measures, using several media campaigns [5], posters, and advertisements on television and printed media along with other methods to improve the awareness of this pandemic among the general population. The assessment of government websites and social media platforms for public awareness is important because it helps determine the impact of governmental prevention efforts and measures and gauges the need for intervention [6].

Researchers have indicated that most developing countries encounter serious difficulties in preventing the spread of infectious diseases due to inadequate medical facilities and lack of resources [7-10]. In light of the apparent weakness of health care systems in these countries, public awareness of infectious diseases leads to behavioral changes among the public, thereby representing partial treatment; notably, this awareness reduces the pressure and economic burden on medical facilities. To raise public awareness, social media platforms are considered to be effective tools that contribute to the real-time dissemination of information about the current status of the disease and give appropriate advice to the public on how to avoid being infected. Further, according to [11], social media platforms provide beneficial climate and socioeconomic data. Additionally, social media platforms have been shown to represent an essential source of communication that enables the creation and dissemination of information to people through the internet [12,13]. It is worth mentioning here that social media platforms allow groups and individuals to exchange information about all subjects and issues, including members of minority groups or people who have no opportunity to express their opinions using other information sources. Researchers have argued that information and perspectives pertinent to issues associated with human health are revealed by informally using social media platforms away from official medical and health departments [14].

Despite the growing body of literature examining social media in health contexts, including public health communication, promotion, and surveillance [1,2], limited insight has been provided into how the utility of social media may vary depending on the particular public health objectives governing an intervention. For example, the extent to which social media platforms contribute to enhancing public health awareness and prevention during epidemic disease transmission is unknown. Korda et al [3] stated:

Evidence about social media’s impact on health knowledge, behavior and outcomes shows that these tools can be effective in meeting individual and population health needs. Most research addresses specific interventions and approaches, which vary widely in focus, target population, theoretical foundations, mode of delivery, functionality, and usability. This wide variation makes it difficult to find out what works and how, and complicates efforts to compare approaches.

Korda et al noted a lack of research focused on the role of social media campaigns on public health awareness of pandemic diseases such as COVID-19, particularly in Jordan. Therefore, in this study, we attempt to answer the following questions:

- Does the use of social media platforms raise public health awareness of COVID-19 as a pandemic disease?
- Does the use of social media platforms increase public behavioral changes toward COVID-19 as a pandemic disease?
- Does the use of social media platforms increase public protection against COVID-19 as a pandemic disease?
- Do public health awareness and behavioral changes play important roles in enhancing the relationship between the use of social media platforms (interventions) and public health protection against COVID-19 as a pandemic disease?

The findings of this study are expected to be helpful and important for public health authorities and governments to understand who receives the intervention (message), the impact of social media platform campaigns, and the extent to which changes in public health behavior and health outcomes can be attributed to the intervention, in addition to determining how the disseminated information is perceived.

### Theoretical Background

Social media platforms are widely deployed by the WHO, health care professionals, and regulatory authorities worldwide to address key issues relating to public health [15]. They can be used to educated citizens and health care professionals on a broad range of themes, from the challenges surrounding anti-microbial resistance to topics such as adverse reaction reporting. The core focus of these initiatives is represented by awareness-building campaigns that take advantage of the large scale, breadth of reach, and immediacy of social media platforms to communicate quickly, effectively, and efficiently. Using social media to aid the prevention and control of infectious diseases can be cost-effective [8]. The health sector represents a critical area of ​​government responsibility in most countries; it accounts for a large proportion of national spending, approximately equivalent to 9.9% of the global gross domestic product in 2016 [16]. Like other segments of the public sector, government health departments and national agencies are responsible for monitoring, protecting, and improving the health of residents, and state-funded health care delivery institutions are under increasing pressure to participate in electronic government (e-government) agendas. Many agencies will likely use social media platforms individually to achieve this. Widespread public engagement with social media platforms creates an effective ready-made path to their application in the health care field.

Social media platforms include a wide variety of networking sites (eg, Facebook), information-disseminating platforms (eg, YouTube), and microblogging services (eg, Twitter). These platforms and many others can be used to create and publish knowledge and information about potential health and disease risks and interventions as well as healthy lifestyles and effective health policies and strategies. In contrast to the campaigns occasionally launched by traditional media, campaigns launched through social media platforms often successfully convert knowledge and information on different health topics into daily fruitful web-based discussions and conversations [17]. Another key advantage of web-based social media data, in addition to the availability of an increasingly large volume of data, is that it is highly contextual and networked [2]. For example, there will be robust spatiotemporal sentiment toward a new vaccine, whether positive or negative. Risk factors such as drug abuse, smoking, poor diet, and lack of exercise and their associated diseases are often found to be clustered in a population. A better understanding of social media platforms and their health data will help broaden the utility of social media in public health.

Social media platforms constitute a powerful means of communication that can be used to elevate public awareness of infectious diseases, particularly new ones, in terms of outbreak dates and spreading developments [18]. Members of the public turn to both traditional and social media to obtain information on emerging infectious diseases which represent unprecedented risks to people [6]. The public perceptions of these risks are shaped depending on how information is communicated across social media platforms. This in turn affects people’s behavior as well as the decisions they make. In addition to information dissemination through social media platforms, the users of these platforms participate in discussions and conversations by giving their own opinions and presenting their own experiences. However, information disseminated through social media platforms often lacks credibility because it is often generated by the users themselves rather than by medical specialists or professional health care institutions; therefore, this information may lack reliability, accuracy, correctness, or usefulness. As a result, the WHO has called for proactive and effective use of social media platforms to disseminate information on health issues, explicitly on emerging infectious diseases, to unspecialized persons and the general public.

### Literature Review

Social media platforms have attracted the interest and attention of researchers and practitioners in the health domain, who use them for different purposes. These include professional training and development of clinicians; formation of health networks and support groups; provision of funding for health institutions; facilitation of cooperation and coordination among health professionals; monitoring of infectious diseases [2].

Even though social media platforms provide professionals in the public health domain with numerous valuable opportunities and benefits, usage of social media platforms by professionals is associated with several challenges, the most important of which are detecting infectious disease outbreaks, monitoring emergencies, predicting disease trends, and measuring the public’s awareness and responses. However, many studies have reviewed and explored the potential applications of social media platforms for public health communication. For example, Huebsch et al [19] suggested that social media platforms allow health practitioners to establish a direct relationship with their clients and that health promotion planners must put forward their creative best to integrate social media platforms within their strategies to make full use of the potential of these platforms when marketing their products and services.

In a study conducted by Bennett and Glasgow [20], they examined the issues of how citizens seek to consume medical information and how the World Wide Web transforms the relationship between the public and medical professionals. On the other hand, Moorhead et al [21] reviewed studies that were conducted to investigate the uses, advantages, and limitations of social media platforms in realizing fruitful communication between health professionals, patients, and the general public. Although the advantages of using social media platforms in health communication were identified in some relevant studies, we noticed a lack of studies that discussed the assessment of the effectiveness of social media platforms in shaping and modifying health communication practices in the short and long terms. The use of social media platforms in disease surveillance was reviewed by Ellis et al [22]. Their literature review revealed the effectiveness of social media platforms in speed, accuracy, and cost performance; therefore, they recommended the use of social media programs to support existing disease surveillance systems. Moreover, a few studies focused on the role of social media platforms in promoting health protection and increasing the relevance of public health messaging, in addition to identifying the lessons learned from social media health campaigns. Among these was an investigation carried out by Collinson et al [23] that indicated the importance of controlling the spread of influenza and reducing the infection effects on a population to public health. Social media campaigns related to epidemics or pandemics can be beneficial in conveying information to the general public, thereby inducing positive attitudes and behaviors that may slow the spread of the disease, such as hand washing and social distancing.

Studies on social media campaigns and healthy behavior have reported that social media campaigns can elicit positive behavior changes and even prevent negative behavior changes in individuals. Social media platforms can be used to reduce the spread of pandemics, thereby lowering the levels of fear and anxiety among the general public. Researchers have argued that social media communication can transfer useful information about infectious diseases based on identifying and tracking users’ behavioral patterns [24]. A model was suggested by Misra et al [7] to explore the impact of awareness realized by social media campaigns on infectious disease prevalence, considering that the campaigns launched on social media platforms represent a variable whose growth depends on the number of individuals infected. The proposed model is based on the assumption that social media health campaigns lead to behavioral changes among individuals, causing them to isolate themselves and protect themselves from infection. The findings of the study by Misra et al [7], referred to above, reveal a decrease in the number of infected people with the increasing spread of social media health campaigns. The models discussed above were extended by Samanta et al [25] by assuming that people who are susceptible to a disease but aware of it are less likely to be infected than people who are unaware.

Further, it was assumed that social media health campaigns grow as the mortality rate of the disease increases. Researchers investigated the role of social media platforms in eliminating infectious diseases in the presence of treatment [14]; they found that information conveyed to the public by social media platforms through health campaigns leads to behavior modification [26]. Another study [27] showed the influence of information related to vaccination against infectious diseases on the endemic state; the results indicated that lower social media coverage leads to a globally stable endemic state. In contrast, the endemic state is characterized by instability and fluctuations in the case of higher social media coverage.

Previous research has found that disease prevalence is controlled by social media coverage. Behavioral changes related to infectious diseases through campaigns launched by print media, social media, and the internet are limited to a smaller population of educated people. In comparison, television advertisements tend to be capable of affecting the behavior toward infectious diseases of a larger population that generally consists of less educated people; therefore, television advertisements are more effective than other types of media. It has been found that social media health campaigns are associated with the number of infected individuals, where launching more campaigns on social media platforms reduces the number of infected people. The effectiveness of interventions made by social media networking sites in modifying the health behaviors of individuals was the subject of an investigation conducted by Laranjo et al [12], who uncovered a positive impact of those interventions on the health behaviors of individuals, but with a remarkable level of heterogeneity. Researchers found that internet data contribute to conveying useful information to the public, supporting the existing health surveillance systems and assisting in the fight against disease [8]. An evaluative examination was carried out [28] in which it was found that during outbreaks of infectious diseases, social media platforms negatively affect the quality of disease prediction and detection. The study was conducted on the diffusion of the Ebola virus as a case study. Sharma et al [29] found that most of the information posted on Facebook about the Zika virus was inaccurate and irrelevant. The influence of social media communication on shaping the behaviors of the general public was the concern of a study [30] about the Ebola and Zika viruses. It was found that users’ trust in both media and authorities represents an important factor in the relationship under study. Most citizens lack accurate and relevant knowledge about the spread of infectious diseases over time and across space. Therefore, social media platforms can be used to establish a database of disease occurrences in terms of time and space. As useful surveillance tools, information on social media platforms was found to outperform official information about the outbreak and spread of infectious diseases, particularly in timeliness.

### The Study Model

Elevating public health awareness was found to require the incorporation of some theories of behavioral change into social media health interventions [31]. According to [32], behavioral change theories (eg, social cognitive theory and health belief theory) can be beneficial when applied to social media initiatives because they help public health authorities understand the process of changing behaviors and why people behave in a precise manner, thereby enabling the health authorities to evaluate and modify health interventions [33-35]. According to the Health Belief Model, people tend to take preventive actions if they feel that they are seriously threatened. Based on this, health interventions should address the specific perceptions of individuals about susceptibility and benefits [36]. The behavioral change approach has been found to improve health by changing people’s lifestyles [37]. It is assumed that individuals must understand basic facts about a specific issue related to their health to be able to change their lifestyles as a result of feeling threatened, especially by an infectious disease. In this context, individuals should learn a group of skills and be granted access to suitable services.

Behavior changes include but are not limited to hand washing, wearing masks, social distancing, avoiding public gatherings, sanitation, and isolation. Interventions aimed at promoting public health can improve the quality of health in society and support the policies and programs run by official health authorities in fighting the outbreak and spread of infectious diseases. If people have trust in these policies and programs, they are likely to respond positively to public health interventions and participate in the launched health promotion programs in large numbers. Social media health campaigns can induce positive behavioral changes and even eliminate negative ones in individuals. According to Laranjo et al [12], the advantages of social media health interventions are cost-efficiency, ubiquity, and passing geographical barriers. The tremendous growth in social media networking sites has opened the door to more opportunities to disseminate health interventions to the general public in real time and irrespective of geographical location, thus leading to public health promotion and positive behavioral changes.

**Figure 1:**
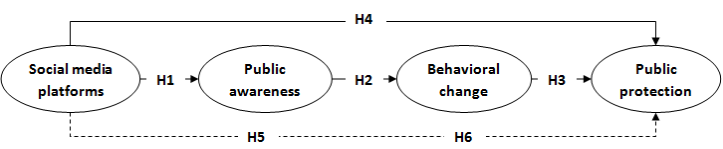
Diagram of the study model.

Therefore, an integrated conceptual model was developed to guide the objectives of this study. It was assumed that social media interventions as tools of health promotion programs would increase public protection and prevention against COVID-19 via the interaction between public awareness and behavior changes as mediating factors. These primary constructs are mainly derived from a theoretical background, relevant previous studies, and behavior change theory (health belief theory). In this study, the variables of primary interest (the independent variables) were social media platforms. The influence of the independent variables on the variance in the dependent variable (public protection against COVID-19) via the mediating factors (public awareness and behavioral changes) was studied. The expected relationships among these constructs are illustrated in Figure 1.

Based upon the above model, the following hypotheses were formulated concerning the role of social media campaigns in increasing public awareness of COVID-19 as a pandemic disease in Jordan:

Hypothesis 1 (H_1_): The use of social media platforms is significantly increasing public health awareness.

Hypothesis 2 (H_2_): Public health awareness is significantly contributing to public health behavioral change.

Hypothesis 3 (H_3_): Public health behavioral change is significantly increasing public health protection.

Hypothesis 4 (H_4_): The use of social media platforms is significantly increasing public health protection.

Hypothesis 5 (H_5_): Public health awareness is significantly mediating the relationship between social media platforms and public health awareness.

Hypothesis 6 (H_6_): Public health behavioral change is significantly mediating the relationship between the use of social media platforms and public health awareness.

## Methods

### Study Design

We employed a quantitative method with an exploratory and descriptive design. To confirm the conceptual model of the research and to investigate the research hypotheses, a survey questionnaire was employed to collect data (Multimedia Appendix 1). The target population of this research consisted of all followers on any social media platform in Jordan. To reach them, a web link to the questionnaire was sent to potential respondents during the period between March 15 and March 30, 2020. The questionnaire was prepared in the Arabic and English languages, and 2555 social media users were sampled to collect the data. The content of the questionnaire (constructs and measures) was mainly selected and adopted from previous relevant studies [2,10,29,30,36-38] using a 5-point Likert scale ranging from strongly disagree (1) to strongly agree (5). Table 1 summarizes these constructs and their related measurement items. To perform construct validation, the questionnaire content was modified to the practice of Jordanian business culture context based on the results of a pilot study and feedback from six professional academic staff members in this field. The survey instrument was reviewed by a panel of six academic researchers in the areas of marketing and management information systems to guarantee face validity. Consequently, several questions were modified, and the revised questionnaire was used for pilot testing on citizens who lived in Jordan during the COVID-19 pandemic. Indeed, a pretest was conducted with 25 citizens to check the understandability of the questions. Some revisions were made, resulting in an easily understandable survey questionnaire.

Table 2 shows that 1283 of the 2555 respondents in this study (50.2%) were female, and 1823 (71.3%) were aged 18 to 44 years. Additionally, 1352 of the 2555 respondents (52.9%) have a bachelor’s degree, and 1219 (47.7%) live in Amman.

**Table 1:** Variables and measurement items.

Construct and measurement items		Description
**Social media platforms (SMP)**		
	SMP1	Facebook helps me to recognize COVID-19^a^.
	SMP2	Instagram helps me to recognize COVID-19.
	SMP3	Twitter helps me to recognize COVID-19.
	SMP4	WhatsApp helps me to recognize COVID-19.
	SMP5	YouTube helps me to recognize COVID-19.
**Public awareness** **(PAW)**		
	PAW1	Facebook contributes to increasing my awareness/knowledge of how to prevent COVID-19.
	PAW2	Instagram contributes to increasing my awareness/knowledge of how to prevent COVID-19.
	PAW3	Twitter contributes to increasing my awareness/knowledge of how to prevent COVID-19.
	PAW4	WhatsApp contributes to increasing my awareness/knowledge of how to prevent COVID-19.
	PAW5	YouTube contributes to increasing my awareness/knowledge of how to prevent COVID-19.
**Public behavioral change (PBC)**		
	PBC1	Facebook contributes to changes in my behavior to prevent COVID-19 by taking various preventive measures (such as not shaking hands or kissing, not leaving the house, eating healthy food and vitamins, general hygiene, lack of anxiety and fear of disease, and increasing religious belief).
	PBC2	Instagram contributes to changes in my behavior to prevent COVID-19 by taking various preventive measures (such as not shaking hands or kissing, not leaving the house, eating healthy foods and vitamins, general hygiene, lack of anxiety and fear of disease, and increasing religious belief).
	PBC3	Twitter contributes to changes in my behavior to prevent COVID-19 by taking various preventive measures (such as not shaking hands or kissing, not leaving home, eating healthy foods and vitamins, general hygiene, lack of anxiety and fear of disease, and increasing religious belief).
	PBC4	WhatsApp contributes to changes in my behavior to prevent COVID-19 by taking various preventive measures (such as not shaking hands or kissing, not leaving home, eating healthy food and vitamins, general hygiene, lack of anxiety and fear of the disease, and increasing religious belief).
	PBC5	YouTube contributes to changes in my behavior to prevent COVID-19 by taking various preventive measures (such as not shaking hands or kissing, not leaving home, eating healthy food and vitamins, general hygiene, lack of anxiety and fear of the disease, and increasing religious belief).
**Public Protection (PPR)**		
	PPR1	Social media platforms contribute to behavioral changes to protect me from infection with COVID-19.
	PPR2	Social media platforms contribute to behavioral changes to protect others from infection with COVID-19.
	PPR3	Social media platforms contribute to behavioral changes in educating others about infection with COVID-19.

^a^COVID-19: coronavirus disease.

**Table 2:** Sample profile (N=2555), n (%).

Characteristic		Value
**Gender**		
	Male	1272 (49.8)
	Female	1283 (50.2)
**Age (years)**		
	18-33	1196 (46.8)
	34-43	627 (24.5)
	44-53	447 (17.5)
	54-63	206 (8.1)
	≥64	79 (3.1)
**Educational level**		
	High school or less	112 (4.4)
	Diploma	262 (10.3)
	Bachelor’s degree	1352 (52.9)
	Master’s degree	421 (16.5)
	PhD	520 (20.4)
**Governorate**		
	Irbid	310 (12.1)
	Balqa	123 (4.8)
	Jerash	37 (1.4)
	Zarqa	158 (6.2)
	Tafilah	17 (0.7)
	Ajloun	29 (1.1)
	Aqaba	276 (10.8)
	Amman	1219 (47.7)
	Karak	227 (8.9)
	Madaba	52 (2.0)
	Maan	39 (1.5)
	Mafraq	68 (2.7)

### Descriptive Analysis

To illustrate the respondents’ attitudes towered each question, they were asked in the assessment, and the mean and SD were accordingly calculated for all the measurements. The descriptive statistics offered in Table 3 point to a positive disposition toward the items measured. The level of every item was calculated by the follow method: (highest point in Likert scale – lowest point in Likert scale)/the number of the levels used = (5 – 1)/5 = 0.80, where a level of 1 to 1.80 was considered very low, 1.81 to 2.60 was low, 2.61 to 3.40 was moderate, 3.41 to 4.20 was high, and 4.21 to 5 was very high. After that, the items were ordered by their means [39,40]. Tables 3 and 4 show the results of the calculations.

**Table 3:** Descriptive statistics of the research items and variables.

Category		Mean (SD)	Level	Order
**Social Media Platforms (SMP)**				
	SMP1	3.84 (1.111)	High	1
	SMP2	3.07 (1.132)	Moderate	5
	SMP3	3.17 (1.133)	Moderate	4
	SMP4	3.54 (1.304)	High	3
	SMP5	3.81 (1.151)	High	2
**Public Awareness (PAW)**				
	PAW1	3.86 (1.117)	High	1
	PAW2	3.17 (1.148)	Moderate	5
	PAW3	3.18 (1.131)	Moderate	4
	PAW4	3.58 (1.251)	High	3
	PAW5	3.71 (1.144)	High	2
**Public Behavioral Changes (PBC)**				
	PBC1	3.94 (1.120)	High	1
	PBC2	3.28 (1.159)	Moderate	4
	PBC3	3.26 (1.139)	Moderate	5
	PBC4	3.67 (1.216)	High	3
	PBC5	3.72 (1.130)	High	2
**Public Protection (PPR)**				
	PPR1	3.99 (1.065)	High	1
	PPR2	3.99 (1.052)	High	1
	PPR3	3.96 (1.067)	High	2

**Table 4:** Overall means, SDs, levels, and orders of the study variables.

Type and variable		Mean (SD)	Level	Order
**Independent**				
	Social media platforms	3.4849 (0.84353)	High	4
**Mediating**				
	Public awareness	3.5011 (0.88427)	High	3
	Public behavioral change	3.5754 (0.90706)	High	2
**Dependent**				
	Public protection	3.9808 (1.02517)	High	1

### Exploratory Factor Analysis

Exploratory factor analysis is often used to gather information about research variables [39]. The outcome of the Kaiser-Meyer-Olkin test was 0.894, and all items were higher than 0.60; consequently, all items were used in the data analysis to capture the investigated latent variables. Furthermore, for the multicollinearity issue, the results indicate that the variance inflation factor for each variable was below 3, suggesting that multicollinearity was not an issue. The findings also indicate the absence of common method bias in that the first factor did not account for the majority of the variance, and no single factor occurred from the factor analysis [41].

### Confirmatory Factor Analysis

**Figure 2:**
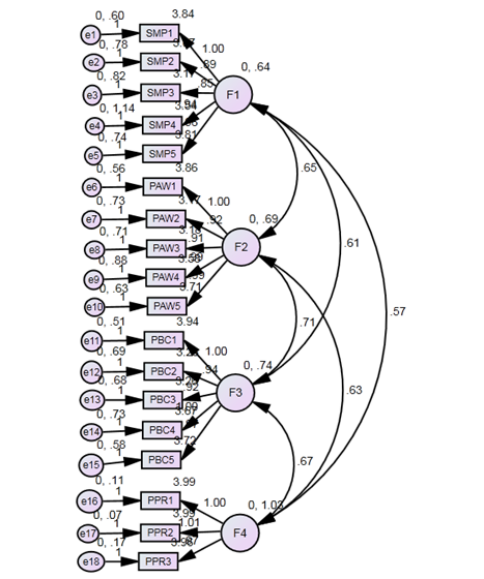
Measurement model showing the correlations among the four research variables.

Confirmatory factor analysis (CFA) was performed to confirm the properties of the research items. Scholars have reported that the measurement model shows how latent variables or hypothetical variables are evaluated under the conditions of observed variables, representing how the validity and reliability of the observed variables answers for the latent variables [42,43]. Figure 2 shows the measurement model and the correlations among the four research variables; it can be seen that all variables were correlated.

The initial CFA model provided an acceptable fit without eliminating any items to achieve an enhanced fitting measurement model. The goodness of fit indices of the evaluation of the initial research model indicated that the findings of the initial model could be deemed as the final model. CFA showed that for the model, χ²_1544_=4281.7 (*P*<.001), which implies that the measurement model fitted the data. Also, χ²/df (4281.742/1544) = 2.773; this is an absolute fit index with a threshold of <3 for a serious viewpoint or <5 for adequate criteria. The incremental fit index of 0.89, the Tucker-Lewis index of 0.86, the comparative fit index of 0.87, and the root mean square error of approximation of 0.052 all meet the threshold of <1 for sufficient criteria [43]. Based on these fit indices, the measurement model indicated a good fit of the sample data.

To determine the reliability and validity of the research model, factor loadings, Cronbach alpha, composite reliability, and average variance extracted (AVE) for the variables were calculated. The entire research indicator (ie, factor loadings) surpassed 0.50 [42,44]; this confirmed the convergent validity. All the composite reliability values surpassed 0.60, representing a high level of internal consistency for the latent variables. Also, each AVE value surpassed 0.50 [43]; thus, the convergent validity was proved (Multimedia Appendix 2 [45]).

## Results

As shown in Table 6, the structural equation modelling analysis showed that H_1_, H_2_, H_3_, and H_4_ were supported.

**Table 6:** Path analysis results for hypotheses 1 to 4. For all hypotheses, *P*<.001.

Hypothesis	Path	Standardized effect (β)	Robust *t* (df)	Result
1	SMP^a^→PAW^b^	.823	64.128 (1544)	Supported
2	PAW→PBC^c^	.704	39.096 (1544)	Supported
3	PBC→PPR^d^	.465	16.134 (1544)	Supported
4	SMP→PPR	.149	5.301 (1544)	Supported

^a^SMP: social media platforms.

^b^PAW: public awareness.

^c^PBC: public behavioral change.

^d^PPR: public protection.

To examine the mediating effects of public awareness, public behavioral change, and social media platforms on public protection, we considered both direct and indirect effects. It was found that public awareness and public behavioral change significantly affected public protection both directly (α=.149 for both H_5_ and H_6_) and indirectly (α_H5_=.571, α_H6_=.579), resulting in total effect sizes of α=.720 and α=.728 for H_5_ and H_6_, respectively; as a result, the data support partial mediation.

**Figure 3:**
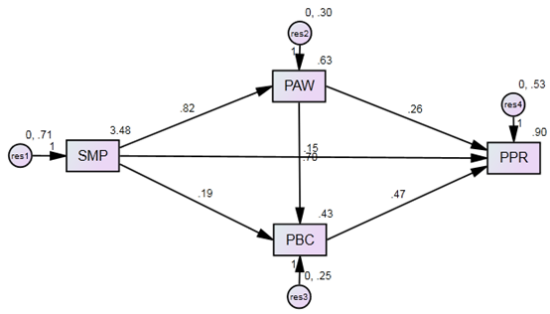
Estimated path values for the hypothesized structural model.

Further, to investigate the structural model, path analysis was conducted. We examined the research hypotheses via the statistical significance of the standardized regression weights (ie, *t* value; see Table 6) and the coefficient of determination R² for the endogenous research variables. The coefficients of determination for public awareness, public behavioral change, and public protection were 0.62, 0.70, and 0.50, respectively; these results show that the model strongly accounts for the variation of the research model. Figure 3 shows the estimated path values for the hypothesized model.

## Discussion

### Principal Findings

In this study, we aimed to explore the impact of using social media platform applications on health and safety during the COVID-19 pandemic through public health awareness and behavioral changes as mediating factors in Jordan. To achieve the study objectives and conduct the research using a systematic approach, a conceptual framework was developed based on a literature review and health belief change theory. The potential benefits of using social media platforms in public health protection against pandemic diseases include dissemination of public health interventions, enhanced public awareness, promotion of healthy behavior, improved health outcomes, and provision of health information to the community [8,24,46-50]. The analysis provides empirical evidence regarding the impact of using social media platforms on public health awareness, public health behavior changes, and health protection against COVID-19 in hypotheses 1, 3, and 4, respectively. These three hypotheses significantly and positively supported the linkage between use of social media platforms and public health awareness, public behavioral changes, and health protection. Numerous research studies have explored the relationship between the use of social media platforms and public health [2,10,12,29,30,36-38,47]. Furthermore, the analysis provided empirical evidence regarding the effectiveness of public health awareness on public health behavioral changes, as proposed in H_4_. The results showed that the effect was positive and significant. Therefore, H_4_ agreed with the findings of Lunn et al [31].

The fifth and sixth hypotheses (H_5_ and H_6_) were developed to determine whether there were mediating effects of public health awareness and public health behavioral changes on the relationship between the use of social media platforms and health protection against COVID-19. The results clearly showed that public health awareness and public health behavioral changes mediated the effects of social media platform use on health protection; however, the mediating effect was partial. Additionally, the results indicated a significant and positive indirect effect of social media platform use on health protection against COVID-19 through public health awareness and public health behavioral changes, with standardized indirect effects of 0.571 and 0.579 and *P* values <.001. The results showed a significant and statistical effect of social platforms use on health protection against COVID-19 without the mediating effects of public health awareness and public health behavioral changes. The total standardized effect was 0.149, which was significant (*P*<.001) but weak.

This discussion shows that the standardized direct effects between social media platform use and public health protection with public health awareness and public health behavioral change as mediators increased to .720 and .728, respectively, and the standardized direct effect of the same relationship in the absence of mediators was .149. This indicates that the mediation was partial. Thus, this result supports H_5_ and H_6_. Moreover, the indirect effects of social media platform use on health protection through public health awareness and public health behavioral change as mediators were both positive and significant, with a standardized indirect effect at a *P* value <.001. Therefore, the mediating effects of public health awareness and public health behavioral changes between usage of social media platforms and public health protection may be a new relationship. The results indicated that social media use had a significant and direct positive effect on public health protection. Social media platform use also had a direct effect on public health awareness; this effect was also significant, as was the effect of public health awareness on public health protection against COVID-19. However, no previous empirical research studies have examined the mediating effects of public health awareness and public health behavioral changes on the relationship between social media use and public health protection.

Therefore, the results confirm that public awareness and public health behavioral changes have vital mediating effects on the relationship between the use of social media platforms and public health protection, and the degree of mediation was partial. This supports H_5_ and H_6_. Furthermore, the main statistical results supported the predictive validity of the conceptual model of the study. Overall, the study validated the use of social media platforms to improve public health protection through public awareness. Therefore, we conclude that social media campaigns should be used to inform the public so that behavior changes can result.

### Theoretical Contributions and Implications

This study fills the gap within the literature regarding a comprehensive understanding of the relationships between social media platform use, public protection against COVID-19 during the pandemic, public awareness, and public health behavioral changes. It also significantly contributes to supporting health belief theory by supporting the links between social media platform use and public protection against COVID-19 via the mediating roles of public awareness and public health behavioral changes. This study provides many theoretical contributions to the literature on social media platform applications for public health care and protection against COVID-19 as a pandemic disease, one of which is to validate the research framework applied in Jordan. Moreover, this research supports the application of social media platforms in Jordan, particularly in public health awareness.

The results endorse the mediating effect of public awareness and public health behavioral changes on the relationship between the independent variable, social media use, and the dependent variable, public health protection, which is another gap addressed by this research. Furthermore, this study has extended the literature that considers social media applications by providing the following:

First, we examined an unexplored connection between social media applications and public health protection against COVID-19 as a pandemic disease. Previous research examined and linked the effects of social media application campaigns on public health awareness and the relationship between public awareness and behavioral health changes.

Second, we validated and tested the impact of the role of public awareness and public health behavioral changes as a mediating factor between the effects of the use of social media applications and public health protection, which can be considered as another contribution to the literature.

The research findings of this study also have significant implications for governmental health officials, health care professionals and practitioners, and other decision makers in health organizations. First, they should be fully aware of the importance of the use of social media campaigns (interventions) for public health awareness and of behavioral health changes to protect their communities and nations from the spread of pandemic diseases such as COVID-19. Second, the role of social media (innervations) to enhance public health awareness and behavioral health changes should be adequately considered in any choice of strategic health promotion plan. Social media campaigns should be considered as critical components of comprehensive approaches to improving public health behaviors.

### Limitations

This study is not without limitations. The application of the conclusions of the study may be limited by time and geographical location. This study is a cross-sectional survey; the underlying identified associations may contrast across divisions and countries or may even lose their meaning over time. Most importantly, these data are self-reported by self-selected participants; also, the lockdown period was a constraint to gathering more representative data.

### Conclusion

Our findings suggest that the use of social media platforms can positively influence awareness of public health behavioral changes and public protection against COVID-19. Public health authorities may use social media platforms as useful tools to increase public health awareness through the dissemination of brief messages to targeted populations. More research is needed to validate how social media channels can be used to improve health knowledge and adopt healthy behaviors in a cross-cultural context.

## Appendix

Multimedia Appendix 1 Survey questionnaire (English version).

Multimedia Appendix 2 Properties of the final measurement model.

## Abbreviations

AVE: average variance extracted
CFA: confirmatory factor analysis
COVID-19: coronavirus disease
e-government: electronic government
H1: hypothesis 1
H2: hypothesis 2
H3: hypothesis 3
H4: hypothesis 4
H5: hypothesis 5
H6: hypothesis 6
SARS-CoV-2: severe acute respiratory syndrome coronavirus 2
WHO: World Health Organization
