# Supplementary material for: Influence of Social Media Platforms on Public Health Protection Against the COVID-19 Pandemic via the Mediating Effects of Public Health Awareness and Behavioral Changes: Integrated Model
Source: J Med Internet Res. 2020 Aug 19;22(8):e19996. doi: 10.2196/19996 (PMC7439806; doi:10.2196/19996)
Supplement: Multimedia Appendix 1 [file jmir_v22i8e19996_app1.docx]

Dear Sir/Madam,

Based on - national and scientific duty towards our country, several researchers from the University of Jordan, Amman & Aqaba Branch; and Princess Sumaya University for Technology are conducting joint scientific research entitled " The Influence of Social Media Platforms on Public Health Protection against Coronavirus (COVID-19) Pandemic Disease via the Mediating Effects of Public Health Awareness and Behavioral Change: An Integrated Model". Therefore, we developed a set of questions in the form of a questionnaire to answer our research questions. Please answer accurately and objectively the questionnaire paragraphs by placing a tick (√) in the appropriate place. This information will be used for scientific research purposes only, thank you for your time and cooperation.

Gratefully,

Researchers:

**Part one:** Demographic factors

**Kindly put a tick** **(✓) next to the appropriate answer**

**A- Gender:**

1. Male 2. Female
**B- Age:**

1: 18 to less than years old 34.

2: 34 to less than 44 years old.

3: 44 to less than 54 years old.

4: 54 to less than 64 years old.

5: 64 and over

**C- The educational level:**

1. High school and less

2: Diploma

3: Bachelor

4: Master

5: PhD

**D- Governorate:**

1. Irbid 2. Balqa 3. Jerash 4. Zarqa 5. Tafilah 6. Ajloun

7. Aqaba 8. Amman 9. Karak 10. Madaba 11. Maan 12. Mafraq

**Part Two:** Please determine the degree of approval or disapproval of the following statements by placing a tick **(✓)** in the appropriate place, which is represented in the following options:

(1) Strongly disagree (2) Disagree (3) neutral (4) Agree (5) Strongly agree

| **F1** | **Social Media Platforms** | **1** | **2** | **3** | **4** | **5** |
| --- | --- | --- | --- | --- | --- | --- |
| 1 | The Facebook application helps in identifying Coronavirus disease (Covid-19) |  |  |  |  |  |
| 2 | The Instagram application helps to identify Coronavirus disease (Covid-19) |  |  |  |  |  |
| 3 | The Twitter application helps to recognize coronavirus disease (Covid-19) |  |  |  |  |  |
| 4 | WhatsApp application contributes to identifying Coronavirus disease (Covid-19) |  |  |  |  |  |
| 5 | YouTube contributes to identifying Coronavirus disease (Covid-19) |  |  |  |  |  |
| **F2** | **Public Awareness** | **1** | **2** | **3** | **4** | **5** |
| 10 | Facebook contributes to increasing my awareness /knowledge to prevent coronavirus (Covid-19) |  |  |  |  |  |
| 11 | Instagram contributes to increasing my awareness/knowledge to prevent coronavirus (Covid-19) |  |  |  |  |  |
| 12 | Twitter contributes to increasing my awareness/knowledge to prevent Coronavirus disease (Covid-19) |  |  |  |  |  |
| 13 | WhatsApp contributes to increasing my awareness /knowledge to prevent coronavirus (Covid-19) |  |  |  |  |  |
| 14 | YouTube contributes to increasing my awareness/ knowledge to prevent coronavirus (Covid-19) |  |  |  |  |  |
| **F3** | **Public Behavioral Change** | **1** | **2** | **3** | **4** | **5** |
| 19 | Facebook contributes to change my behaviour to prevent Coronavirus (Covid-19) by taking various preventive measures (such as not shaking hands and kissing, not leaving the house, eating healthy food and vitamins, general hygiene, lack of anxiety and fear of disease, and increasing religious belief) |  |  |  |  |  |
| 20 | Instagram application contributes to change my behaviour to prevent Coronavirus (Covid-19) by taking various preventive measures (such as not shaking hands and kissing, not leaving the house, eating healthy foods and vitamins, general hygiene, lack of anxiety and fear of disease, and increasing religious belief) |  |  |  |  |  |
| 21 | Twitter application contributes to change my behaviour to prevent Coronavirus (Covid-19) by taking various preventive measures (such as not shaking hands and kissing, not leaving home, eating healthy foods and vitamins, general hygiene, lack of anxiety and fear of disease, and increasing religious belief) |  |  |  |  |  |
| 22 | WhatsApp application contributes to change my behaviour to prevent coronavirus (Covid-19) by taking various preventive measures (such as not shaking hands and kissing, not leaving home, eating healthy food and vitamins, general hygiene, lack of anxiety and fear of the disease, and increasing religious belief) |  |  |  |  |  |
| 23 | The YouTube application contributes to change my behaviour to prevent coronavirus (Covid-19) by taking various preventive measures (such as not shaking hands and kissing, not leaving home, eating healthy food and vitamins, general hygiene, lack of anxiety and fear of disease, and increasing religious belief) |  |  |  |  |  |
| **F4** | **Public Protection** | **1** | **2** | **3** | **4** | **5** |
| 28 | Social media platforms contribute to a behavioural change to protect me from infection with Coronavirus (Covid-19) |  |  |  |  |  |
| 29 | Social media platforms contribute to a behavioural change to protect others from infection with Coronavirus (Covid-19) |  |  |  |  |  |
| 30 | Social media platforms contribute to a behavioural change in educating others about infection with Coronavirus (Covid-19) |  |  |  |  |  |

Thank You so Much
