# Supplementary material for: Influence of Social Media Platforms on Public Health Protection Against the COVID-19 Pandemic via the Mediating Effects of Public Health Awareness and Behavioral Changes: Integrated Model
Source: J Med Internet Res. 2020 Aug 19;22(8):e19996. doi: 10.2196/19996 (PMC7439806; doi:10.2196/19996)
Supplement: Multimedia Appendix 2 [file jmir_v22i8e19996_app2.docx]

**Supplementary table: Properties of the final measurement model**

| Variables and  Items | Factor Loadings | | Std.  Error | Square  Multiple  Correlation | Error  Variance | Cronbach  Alpha | Composite  Reliability* | AVE** |
| --- | --- | --- | --- | --- | --- | --- | --- | --- |
|  | EFA | CFA |  |  |  |  |  |  |
| SMP |  |  |  |  |  | 0.770 | 0.89 | 0.90 |
| SMP1 | 0.716 | 0.719 | *** | 0.517 | 0.20 |  |  |  |
| SMP2 | 0.604 | 0.625 | 0.030 | 0.391 | 0.24 |  |  |  |
| SMP3 | 0.600 | 0.603 | 0.030 | 0.363 | 0.25 |  |  |  |
| SMP4 | 0.601 | 0.609 | 0.034 | 0.329 | 0.34 |  |  |  |
| SMP5 | 0.658 | 0.665 | 0.030 | 0.442 | 0.23 |  |  |  |
| Public Awareness |  |  |  |  |  | 0.821 | 0.91 | 0.93 |
| PAW1 | 0.772 | 0.744 | *** | 0.554 | 0.17 |  |  |  |
| PAW2 | 0.676 | 0.666 | 0.027 | 0.444 | 0.22 |  |  |  |
| PAW3 | 0.672 | 0.666 | 0.027 | 0.443 | 0.21 |  |  |  |
| PAW4 | 0.689 | 0.660 | 0.029 | 0.435 | 0.26 |  |  |  |
| PAW5 | 0.732 | 0.717 | 0.027 | 0.514 | 0.19 |  |  |  |
| Public Behavioral Change |  |  |  |  |  | 0.846 | 0.92 | 0.93 |
| PBC1 | 0.778 | 0.768 | *** | 0.590 | 0.17 |  |  |  |
| PBC2 | 0.691 | 0.696 | 0.026 | 0.485 | 0.21 |  |  |  |
| PBC3 | 0.688 | 0.691 | 0.025 | 0.478 | 0.21 |  |  |  |
| PBC4 | 0.725 | 0.710 | 0.027 | 0.505 | 0.23 |  |  |  |
| PBC5 | 0.738 | 0.737 | 0.025 | 0.544 | 0.18 |  |  |  |
| Public Protection |  |  |  |  |  | 0.964 | 0.98 | 0.94 |
| PPR1 | 0.798 | 0.952 | *** | 0.907 | 0.05 |  |  |  |
| PPR2 | 0.799 | 0.969 | 0.009 | 0.939 | 0.04 |  |  |  |
| PPR3 | 0.791 | 0.923 | 0.010 | 0.853 | 0.06 |  |  |  |
| Instrument Total |  | | | | | | | |
| Cumulative (%) | 76.502 |  |  |  |  |  |  |  |
| KMO | 0.894 |  |  |  |  |  |  |  |
| P-value | 0.000 |  |  |  |  |  |  |  |

* Employing [45]'s formula, the following equation expresses the composite reliability calculation:

Composite Reliability = (ΣL*i*) ² / ((Σ*Li*) ² + ΣVar (E*i*))

*where Li is the standardized factor loadings for each indicator, and Var (Ei) is the*

*error variance associated with the individual indicator variables.*

** The formula for the variance extracted is:Average Variance Extracted = ΣL*i* ² / (Σ*Li* ² + ΣVar (E*i*))

*where Li is the standardized factor loadings for each indicator, and Var (Ei) is the*

*error variance associated with the individual indicator variables.*
